# Current Knowledge of and Perspectives about the Pathogenesis of Blood Blister-like Aneurysms of the Internal Carotid Artery: A Review of the Literature

**DOI:** 10.7150/ijms.53154

**Published:** 2021-03-03

**Authors:** Xiao-Dong Zhai, Peng Hu, Chuan He, Yue-Shan Feng, Gui-Lin Li, Hong-Qi Zhang

**Affiliations:** 1 Department of Neurosurgery, Xuanwu Hospital, Capital Medical University, Beijing, China.; 2 China International Neuroscience Institute (China-INI), Beijing, China.

**Keywords:** Blood blister-like aneurysms, Internal carotid artery, Pathogenesis, Intracranial aneurysms.

## Abstract

Blood blister-like aneurysms (BBAs) are rare and usually appear at nonbranching sites in the supraclinoid portion of the internal carotid artery (ICA). Because it is difficult to obtain histological specimens of the aneurysm wall and because experimental models are challenging to establish, the pathogenesis of BBAs remains uncertain. In this paper, we reviewed the diagnostic, radiological, and pathophysiological characteristics of patients with BBAs. We also summarized the existing evidence and potential mechanisms related to the causes of BBAs. Current evidence indicates that atherosclerosis and dissection are the main prerequisites for the formation of BBAs. Hemodynamics may play a role in the process of BBA formation due to the unique vascular anatomy of the supraclinoid ICA. Further research on histopathology and hemodynamics is warranted in this field.

## Introduction

Blood blister-like aneurysms (BBAs) usually appear at the anteromedial or anterior wall of the supraclinoid segment of the internal carotid artery (ICA) 1-4. Although BBAs are rare, comprising approximately 0.3% to 1.0% of intracranial aneurysms (IAs), 0.5 to 2.0% of ruptured IAs and 0.9% to 6.5% of ICAs, their propensity to cause spontaneous subarachnoid hemorrhage (SAH) has led to high morbidity and mortality 5-7. Many treatment strategies have been reported in the literature, but the management of BBAs has proven to be intractable, and the optimal therapeutic strategy for BBAs is still under debate 5-12. The microsurgical treatments for BBAs include direct clipping, wrapping, wrap-clipping, trapping, and revascularization. Endovascular therapy can be performed with single coils, stent-assisted coils, multiple stents, and flow diversion (FD) 5, 9, 10, 12-14. During the process of separating, exposing, or clipping the BBA, the aneurysm's neck is easily torn and can cause intraoperative bleeding, leading to a poor prognosis 13, 15, 16. The tortuous supraclinoid portion of the ICA, the fragile aneurysm wall and the small size of the aneurysm result in a lack of stability and support, which are needed for microcatheter and coiling control during endovascular treatment 7, 10, 11, 17.

BBAs typically have a thin, fragile wall and unidentifiable neck 3, 4, 18. In contrast to saccular aneurysms, these lesions show loss of the internal elastic lamina (IEL), vascular intima and media, sometimes appearing as only a fragile fibrous layer 2, 3, 6, 18-20. Under the impact of blood flow, some BBAs can enlarge from a small protrusion to a saccular aneurysm in a short time, with a high risk of rebleeding 1, 4. Due to the difficulty of obtaining histological specimens of the aneurysm wall, the pathogenesis of BBAs remains uncertain. This work aimed to review the current literature to summarize the existing evidence and potential mechanisms related to the causes of BBAs.

## Diagnosis of BBAs

In clinical practice, patients are usually diagnosed with BBAs due to the acute symptoms caused by SAH, and they typically have no complaints until rupture occurs 6, 21, 22. BBA is frequently seen in females, younger individuals, and patients with hypertension 5, 6. These lesions are generally small in size and therefore do not cause symptoms of nerve compression before rupture.

From the digital subtraction angiography (DSA) images, a typical BBA is usually observed as a small irregular hemispherical protrusion on the anteromedial wall of the supraclinoid ICA. Sometimes, BBAs may be accompanied by dissection of the ICA; the lumen of the parent artery can be narrowed or dilated during angiography 2, 18, 23. Due to their small dimensions and atypical morphological features, BBAs are often not easily detected on initial angiography 1, 24. Therefore, three-dimensional angiography detection should be routinely performed, which is conducive to improving the detection rate of BBAs.

The diagnosis of BBAs should not be confirmed only by radiological images and clinical symptoms. Although the imaging manifestations of an aneurysm and the patient's clinical symptoms are consistent with the characteristics of BBAs, the pathological manifestations may be a true aneurysm 20, 22. Kim et al.^20^ reported a patient with SAH who had a small aneurysm at the ICA medial wall on initial angiography. Follow-up angiography 12 days later demonstrated an increased aneurysmal size and a change in morphology to a saccular shape. A histologic examination of the aneurysm wall revealed it as a true aneurysm. Zhao et al. 22 reviewed 43 small broad-necked aneurysms at the supraclinoid segment of the ICA in 41 patients who had been treated with microsurgery. The diagnosis of BBAs and non-blister aneurysms was obtained from the intraoperative inspection. Eventually, only 17 aneurysms were diagnosed as BBAs. In the strict sense, the main diagnostic criteria of BBAs should be determined by histopathological examination, with the absence of the IEL and media 4, 20, 22. Nevertheless, it is challenging to obtain histological specimens of BBAs due to their small size and fragile wall. Therefore, the widely accepted diagnosis of BBAs is based on intraoperative inspection, during which surgeons can even observe the extremely friable aneurysmal wall.

## Treatment of BBAs

Although numerous treatment strategies have been proposed, including microsurgery, endovascular treatment, and combined options, the optimal therapeutic strategy for BBAs is still under debate. Wrapping only has a limited effect on preventing postoperative re-rupture, and direct clipping carries a high risk of intraoperative rupture and subsequent ICA sacrifice 5, 12. The direct clipping technique has not been considered a first-line treatment for the increased risk of complications 5. Due to the small size of BBAs, the clips are unstable and may be dislocated from the original location. Wrap-clipping has the advantage of preserving the anterograde blood flow of the ICA and protecting the weakened aneurysm wall; however, they are at risk for postoperative ICA stenosis or occlusion 12, 13, 25. Extracranial-intracranial high-flow bypass has the potential to cause ischemic complications and graft thrombosis for these patients 15, 26-28.

From the endovascular treatment perspective, the small size and unidentifiable broad-based neck of BBAs limit the effectiveness of coil embolization 5, 29, 30. Stent-assisted coil embolization and flow diversion implantation offer low aneurysm obliteration rates in the acute phase 10, 11, 30-32. Mokin et al. 33 reported that 42% (19/45) of BBAs treated by FD showed partial filling, and only 27% (12/45) showed no residual aneurysm filling at the end of the procedure. Although willis-covered stent implantation showed safe and effective clinical outcomes, ophthalmic artery (OA) or anterior choroidal artery (AchA) occlusion was observed in some patients postoperatively 34. Overall, ruptured BBAs of the ICA are challenging to treat both microsurgically and endovascularly.

## Differences exist in the pathophysiological mechanisms of BBAs and saccular intracranial aneurysms (sIAs)

Saccular intracranial aneurysms (sIAs) are pathological pouch-like dilatations of the intracranial arteries. Although BBAs can grow from a small protrusion to a saccular shape in a short period, the pathological features of BBAs are different from those of sIAs.

Under normal physiological conditions, the intracranial artery usually consists of three layers 35-38. The inner layer is the intima with endothelial cells; the middle layer is mainly composed of smooth muscle cells (SMCs), which are embedded into a dense network of collagen and elastin fibers; and the adventitia mainly consists of collagen, which can provide structural integrity to the artery wall 37, 38.

Compared with the rarity of BBAs, sIAs are a common disease, with a prevalence of 5%-7% in the general population 37, 39, 40. By establishing animal models of aneurysms or analyzing human histological specimens, the predominant model for sIAs formation and progression is obtained 35, 41, 42. First, abnormal hemodynamic stress leads to vascular endothelial cell degeneration 43, 44. Inflammation is considered the leading factor in the pathogenesis of sIAs 45, 46. The loss of tight junction proteins between endothelial cells and the release of proinflammatory cytokines by endothelial cells leads to infiltration of a large number of inflammatory cells, causing SMCs proliferation, apoptosis, and chronic remodeling of the vascular wall 37, 45, 47-49. The degenerated aneurysmal wall becomes too fragile to resist hemodynamic stress; as apoptosis of the IEL and SMCs and the disorganized extracellular matrix (ECM) process gradually increases, sIAs formation and growth occurs, and the aneurysm finally ruptures 35, 41, 42.

**Figure 1:**
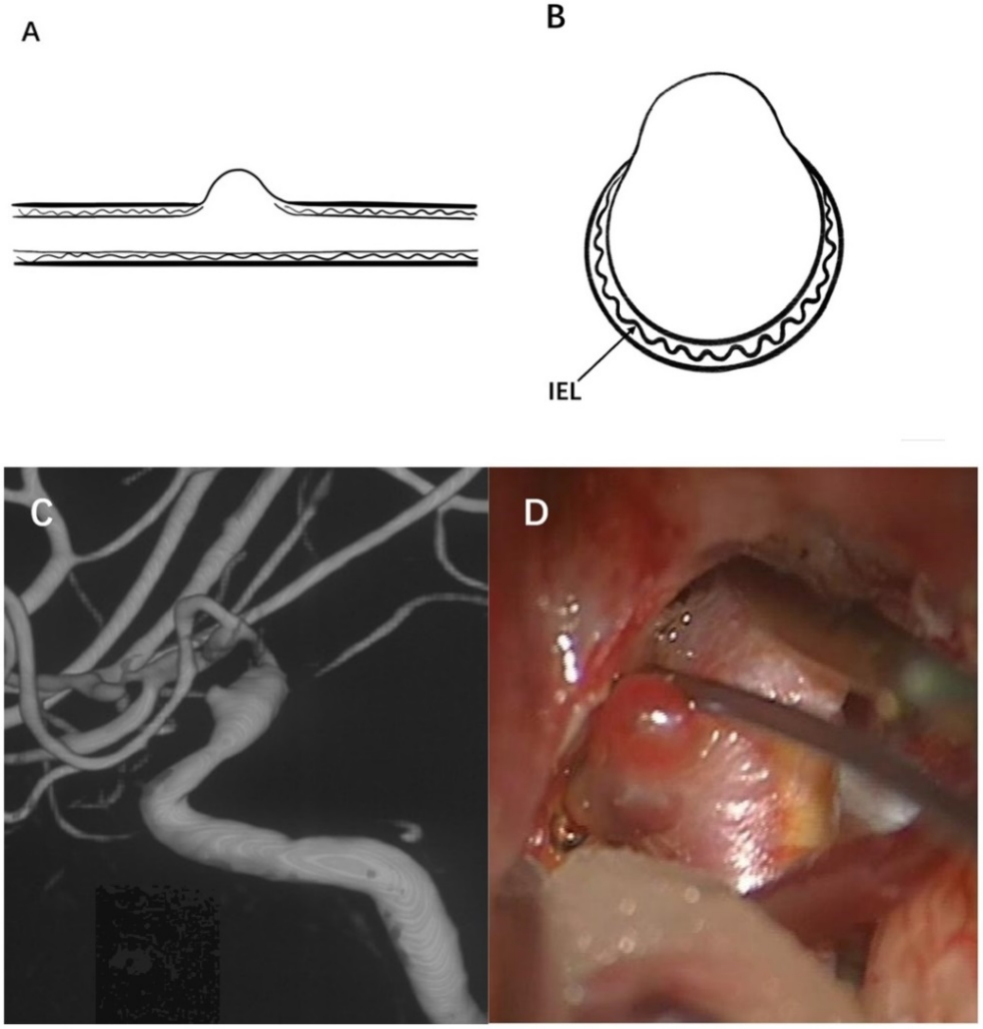
Illustration of a typical BBA. (A) and (B) show the internal elastic lamina, vascular intima and media have disappeared on the aneurysm wall, and the aneurysm is only covered by a fragile fibrous layer. (C) A BBA located on the right supraclinoid ICA is shown, (D) A thin and transparent aneurysm wall was observed during microsurgery.

Therefore, a true aneurysm is formed by the gradual degeneration of the artery wall, and there are usually one or two layers of typical arterial structures in the aneurysm wall. BBAs cannot be considered an early stage of saccular aneurysm development. BBAs do not have a complete arterial wall and lack the IEL and media instead of having thin adventitia and fibrous tissue 50. Moreover, no inflammatory cell infiltration is observed in the wall of BBAs 2. Based on these characteristics, they are considered pseudoaneurysms. Figure 1 illustrates the typical angiographic and intraoperative features of a BBA of the ICA.

## Pathophysiological characteristics of BBAs

BBAs usually show a thin, fragile wall and unidentifiable neck from the intraoperative view; these features make it challenging to obtain a specimen of the aneurysm wall, and experimental models are difficult to establish 18. There are currently limited studies that have reported BBA's pathophysiology, and the sample size is usually small. Therefore, this substantially limits our comprehensive understanding of the mechanisms of the formation and progression of BBAs.

Dissection weakens the artery wall, making it unable to resist hemodynamic stress and eventually leading to rupture of the artery wall 4, 18. Ogawa et al. 1 observed significant ICA dissection in 25% (10/40) of BBAs on angiography or during microsurgery. Sometimes, BBAs are not easily detected on initial angiography but usually show enlargement from a small hemispherical protrusion to a saccular, strawberry-shaped aneurysm in a short time. There are even studies that indicated spontaneous healing of ruptured BBAs 24, 51. Zeineddine et al. 51 reported a case of ruptured BBA along the dorsal surface of the left ophthalmic segment. Repeat angiography two days later demonstrated receding of the ectasia, and no residual abnormality six weeks later. Due to the small size of dissection-related BBAs, focal acute arterial spasm in combination with the impact of blood flow might lead to spontaneous healing. The above evidence suggests that dissection of the artery wall is one of the fundamental prerequisites for BBAs.

Atherosclerotic remodeling, degeneration of the artery wall and loss of the IEL lead to the formation of BBAs 2, 4, 23. Even though patients were relatively young, the cases reported by Sim et al. 23 suggested that all ten patients in their study had atherosclerotic parent arteries adjacent to the BBAs. Ishikawa et al. 2 reported that the ICA wall's arteriosclerosis was observed from an autopsy, and the IEL and media disappeared between the normal and sclerotic ICA walls. These pathophysiological findings, suggest that atherosclerosis causes degeneration of the IEL in the arteries and eventually leads to rupture of the arterial wall. Ulceration associated with atherosclerotic plaques can lead to the destruction of the IEL in the vessel wall and allow hematoma formation within the media of the artery wall, leading to the formation of BBAs.

There exist only a few reports of traumatic-related BBAs. Haji et al. 52 reported one case of cranial trauma with diffuse basal SAH. The computerized tomography angiography (CTA) demonstrated a small, 2-mm BBA from the left supraclinoid ICA's dorsal surface at a nonbranching site. The pathology of the native ICA was observed during microsurgery, raising the possibility of a preexisting BBA. Segmental arterial mediolysis (SAM) is a rare nonatherosclerotic, noninflammatory vascular disease 53. Pickup et al. 54 suggested SAM to be a condition found in Ehlers-Danlos type IV. A small number of SAM-related BBA cases have been reported, which manifest as lysis of the tunica media, smooth muscle degeneration and serration of the lamina elastica interna 55, 56.

## Hemodynamic characteristics

sIAs commonly arise at the bifurcations of the cerebral arteries. In contrast, BBAs usually appear at nonbranching sites in the surpraclinoid portion of the ICA 1, 18, 22. A number of scholars have argued that hemodynamics may play an important role in the formation of BBAs. The hemodynamic characteristics caused by the unique vascular anatomy of the carotid siphon and the formation of BBAs may have some degree of correlation. To our knowledge, the current studies were not carried out with sufficient evidence from hemodynamic studies to draw corresponding conclusions on the mechanisms of BBA formation.

The large curvature of the carotid siphon is one of its essential characteristics. In a computational fluid dynamics (CFD) simulation study, high curvature tightness resulted in a significantly higher wall shear stress (WSS) on the outer wall than on the inner wall of the bend 57. Besides, Meng et al.^43^ analyzed the morphological and hemodynamics characteristics of aneurysms, suggesting that a high WSS is significantly correlated with a small thin-walled and entirely translucent aneurysm. Despite similar appearances, further hemodynamic studies that provide more direct evidence are required to clarify this presumption.

The OA is one of the main branches of the paraclinoid ICA. The branching vessels can affect the hemodynamic characteristics of the ICA trunk through shunting. Indo et al. 58 indicated that ICA anterior wall aneurysms in patients with an OA anomalous origin tend to be saccular aneurysms with normal neck walls. On the other hand, of the ten patients who underwent microsurgery, all six aneurysms with a normal OA showed dissecting aneurysms or BBAs, not saccular aneurysms. It is necessary to conduct further hemodynamic analyses in the future to help explore the pathogenesis of BBAs.

## Conclusion

In this study, we reviewed the diagnostic, radiological, and pathophysiological characteristics of patients with BBAs. We also summarized the existing evidence and potential mechanisms related to the causes of BBAs of the supraclinoid ICA. Current evidence indicates that atherosclerosis and dissection are the main prerequisites for the formation of BBAs. Hemodynamics may play a role in the process of BBA formation due to the unique vascular anatomy of the supraclinoid ICA. Overall, the pathogenesis of BBAs is not entirely clear and further research on their histopathology and hemodynamics is warranted.

## Abbreviations

BBAs: blood blister-like aneurysms
ICA: internal carotid artery
IAs: intracranial aneurysms
SAH: subarachnoid hemorrhage
IEL: internal elastic lamina
DSA: digital subtraction angiography
CTA: computerized tomography angiography
OA: ophthalmic artery
AchA: anterior choroidal artery
SMCs: smooth muscle cells
sIAs: saccular intracranial aneurysms
ECM: extracellular matrix
SAM: segmental arterial mediolysis
CFD: computational fluid dynamics
WSS: wall shear stress
